# Impact of training on knowledge, confidence and attitude amongst community health volunteers in the provision of community-based palliative care in rural Kenya

**DOI:** 10.1186/s12904-024-01415-5

**Published:** 2024-04-11

**Authors:** Hussein Elias, Evelyne Kisembe, Sarah Nyariki, Ivan Kiplimo, James Amisi, Juli Boit, Allison Tarus, Naseem Mohamed, Kenneth Cornetta

**Affiliations:** 1 Department of Family Medicine, College of Health Sciences, Moi University, Eldoret, Kenya; 2 Academic Model Providing Access to Healthcare (AMPATH), Eldoret, Kenya; 3 Living Room International Hospital, Eldoret, Kenya; 4 Webuye County Referral Hospital, Webuye, Kenya; 5 Department of Medical and Molecular Genetics, School of Medicine, Indiana University, Indianapolis, IN USA

**Keywords:** Community health volunteers, Community based palliative care, Training, Curriculum, Teleconsult

## Abstract

**Objectives:**

Existing literature suggests multiple potential roles for community health volunteers (CHVs) in the provision of palliative care (PC) in low- and middle-income countries. In Kenya the role of CHV in the provision of PC has not been reported. The objective of this study was to assess knowledge, confidence, attitude, and clinical practice of community health volunteers after attending a novel palliative care (PC) training program.

**Methods:**

A total of 105 CHVs participated in a 3-day in person training followed by a 1-month in person and telephone observation period of the palliative care activities in the community. Structured questionnaires were used pre- and post-training to assess knowledge acquisition, impact on practice, and content delivery. A mixed method study design was conducted 12-month post training to assess impact on clinical practice.

**Results:**

Immediately after training, CHV provided positive ratings on relevance and content delivery. In the month following training, CHVs evaluated 1,443 patients, referred 154, and conducted 110 and 129 tele consults with the patients and PC providers respectively. The follow up survey at 12 months revealed improved knowledge and confidence in various domains of palliative care including symptom and spiritual assessment and provision of basic nursing and bereavement care. Focus group discussions revealed the CHVs ability to interpret symptoms, make referrals, improved communication/ interpersonal relationships, spiritual intervention, patient comfort measures and health care practices as newly learned and practiced skills.

**Conclusions:**

We noted improved knowledge, new skills and change in practice after CHVs participation in a novel training curriculum. CHVs can make important contributions to the PC work force and be first line PC providers in the community as part of larger hub and spoke care model.

**Supplementary Information:**

The online version contains supplementary material available at 10.1186/s12904-024-01415-5.

## Background

In 2014, the World Health Assembly WHA 67.19 called upon the World Health Organization (WHO) and Member States to improve access to palliative care (PC), with an emphasis on primary healthcare and community/home-based care [1]. Approximately 800,000 people in Kenya are in need of PC while only 14,552 accessing the service [2]. Similar to other countries in Africa, the need of PC in Kenya is escalating with the increasing burden of noncommunicable diseases and the ageing of the population [3–5].

In Kenya most patients are diagnosed in advanced stages of disease and many who present early cannot afford or lack access to curative treatments [5, 6]. Also, an increase in life expectancy and decrease in communicable diseases have led to an increase in chronic disease, such as heart failure and chronic obstructive pulmonary disease putting additional strain on the limited available PC resources [6]. 

In Kenya, Community Health Volunteers (CHV) receive limited training (324 contact hours and 160 h of practical experience), and are then assigned to provide services to a community unit by the local Ministry of Health (MOH) [7]. On average, 10 CHVs are assigned to a community unit of approximately 1,000 households (5,000 people) within a defined geographical area [7–9]Since the 1980s, CHVs have played a major role in the delivery of community-based healthcare in rural areas with activities focused on (a) home visits to determine health situations, deliver key messages and undertake necessary actions, (b) health improvement and prevention, (c) treatment of common diseases and minor injuries, (d) protocol implementation for maternal and newborn health efforts, and (e) case management of childhood illnesses [7–9].

Existing literature indicates multiple potential roles for Community Health Volunteers (CHVs) in the provision of PC in low- and middle-income countries (LMIC) [10]. The scope of practice can vary in different LMIC’s, some of which have shown the potential of CHVs to raising awareness and identifying patients in need of PC, assist in pain management, and provide home-based care including psychological and spiritual support [10, 11]. In Kenya, the role of CHV in the provision of PC has not been reported. One of the emerging themes in a recently published qualitative study on Kenyan palliative care providers and leaders perception of palliative care research needs and support to facilitate rigorous research was to evaluate the role of CHVs in the provision of PC [12].

There is paucity of local data regarding CHVs and palliative care. This project aimed to train CHVs in community based palliative care and equip them with tools to assist them to identify, assess, support and refer community members in need of palliative care. Hence, the broad objective of this study was to assess knowledge, confidence, attitude and clinical practice after attendance at the training program.

## Methods

We developed a curriculum focused on key PC skills and included connections to PC providers within the county of residence. The training involved two components, a 3-day classroom training including practicum and a one-month observation in the community. Because of their limited medical background, CHV were linked to palliative care providers for teleconsultation and facilitate referrals to palliative care clinics. Providers included a primary care physician and clinical officers (equivalent to physician assistant who have prescription authority). Providers have more than 3 years of working experience in a palliative and hospice care unit.

### CHV Selection

The training was developed in collaboration with county health officials from Uasin Gishu and Bungoma counties, Kenya. As the course was delivered prior to widespread COVID19 vaccinations, the officials requested the inclusion of COVID19 education in the curriculum and required training session be limited to < 12 CHV. The county officials selected 2 CHV from a community unit within their jurisdiction. CHVs were invited from 43 community units.

### Training Implementation

The curriculum was developed by PC providers at Moi University School of Medicine and Academic Model Providing Access To Healthcare (AMPATH www.ampathkenya.org), Eldoret Kenya and aimed to teach PC skills appropriate for the CHV scope of practice. The Knowledge Based Curriculum Components are listed in Table 1. Training included a three-day in person combination of didactic and practicum learning followed by a one-month observation period. The training was delivered by experienced clinical officers and nurses working in PC facilities within the county. The training was supervised by a primary care physician. The in-person training was hosted at the hospitals where the trainers work. The skill-based components were delivered using role play. For the observation period, CHVs were provided with a training manual, patient assessment forms, telephone contacts of the trainers and 400 Kenya Shillings (kshs) (approximately $4 US) airtime for the month. Participants were linked to a PC provider within their region who during the observation period met with them weekly in the community to review symptom assessment forms and document activities. In addition, the PC providers visited the households with the CHVs and observed the CHV encounters with the community members. The observation was also done remotely in between the weekly visits using a telephone. The CHVs were called by the trainers and asked about the encounters with the community members and use of the tools. The PC providers were also provided with 800 kshs airtime for the month. A total of 10 training sessions were conducted between August 5th, 2020 and April 6th 2021, with 105 CHVs completing the 3-day training.

**Table 1:** Training components

Knowledge based curriculum components	
**1**	Introductory module on what is palliative care
**2**	The caregiver’s responsibilities to patients/community participation in palliative care (includes ethical principles)
**3**	Socioeconomic and emotional issues
**4**	Physical problems: knowledge, skills and attitude to assess common physical symptoms and apply non-medical interventions
**5**	Spiritual issues
**6**	Knowing how to communicate to the patients in a supportive way
**7**	Knowing the basics of nursing care: prevention of bedsore, sterilizations, asepsis, cleaning and dressing of wounds, stoma care
**8**	Grief and bereavement
**9**	Basics on COVID 19
**10**	Basics of cervical and breast cancer screening
**Skill based curriculum components**	
**1**	Support and educate patients and their families on patient bathing
**2**	Support and educate patients and families on how to feed the patient
**3**	Support and educate the family on how to turn a bedbound patient
**4**	Support and educate the family on pressure area care on the patient
**5**	Support and educate the family on toileting for the patient
**6**	Provide medicine adherence counselling to patients
**7**	Support and educate patients and families on passive and active exercises
**8**	Support and educate family on mouthcare for a comatose or disabled patient
**9**	Provide bereavement support to the family
**10**	Facilitate tele consult with the PC team
**11**	Provide linkage and referral of patients to healthcare facilities

### Curriculum Assessment

Efforts to document the impact of training were conducted at two timepoint. An initial assessment was performed at the time of training. Pre- and post-questionnaires assessed trainees on confidence, relevance, and content delivery. Approximately one year after training, all trained CHV were invited to participate in a mixed method designed study to assess knowledge retention, impact on practice, and challenges to PC delivery. Participation was voluntary, 79 out of 105 trained CHVs chose to participate in the survey and FGDs. The survey was carried out at the sites where the original training was conducted. The focus group discussions were conducted in groups of ≤ 12 participants. A total of 10 FGDs were held. The participants in a training cohort were selected from the participants in the original training cohort. All the participants who accepted and signed the informed consent were asked to complete a self-administered survey and participate in a focus group discussion (FGD). Data collection was designed using Kirkpatrick taxonomy [13] (Table 2). Level 1 Reaction: participants reaction to the training was assessed using the anonymized information from post-training evaluation taken at the time of initial training. Level 2 Learning: acquired knowledge was assessed 12 months after the using Likert type questions derived from the Knowledge Based Curriculum Components. Participants were also asked to rate their confidence level on performing clinical skills. Level 3 Behavior: 12 months post-training a structured questionnaire and FGD assessed how training changed CHV practice. Level 4 Results: at 12 months a questionnaire and FGD evaluated (a) CHVs confidence in their ability to identify patients in need of PC, (b) ability to refer palliative patients, (c) cope with the very sick patients and (d) provide home-based care and (e) train other CHVs. The CHVs were also questioned regarding their use of the training manual and the assessment forms. The number of consultation requests from the community where the trained CHVs practiced was also assessed. The questionnaires used were developed for this study (see supplementary file 1).

**Table 2:** Time of assessment

Kirkpatrick taxonomy level	Type of assessment	Time of assessment
I (Reaction)	Post training evaluation survey	After completion of the 3 day in person training
II (Learning)	Acquired knowledge and confidence assessment	12 months after completion of the 3 day in person training
III (Behavior)	Structured questionnaire and FGDs	12 months after completion of the 3 day in person training
IV (Results)	Structured questionnaire and FGDs	12 months after completion of the 3 day in person training

Data from the survey was extracted into Redcap for compiling and reviewing. All the data was anonymized, and stored in a password protected server. The FGD interviews were held in English and audio-recorded. The data was transcribed using NVivo software for coding into meaning oriented interpretations. In instances where the participants chose to speak in Swahili, the data analyst translated the wording into English. Statistical analysis of pre- and post- questionnaires was performed using two-tail paired t-test.

The data was maintained under Institutional Research and Ethics Committee (IREC) guidelines to respect confidentiality as data moved through the stages of capture, storage, entry, cleaning, coding, analysis. (A) Data was stored in files using a naming convention to maintain confidentiality of the participants by delinking identifiers. The naming conventions identifies file content without opening the file. The naming conventions carried four components: study phase, participant group, participant ID, file type; (B) Data and recording files were saved in a password protected computer and stored in a locked cabinet when not in use by study personnel.

The study was approved by the Moi University IREC (IREC 2021/203) and was determined to be exempt by Indiana University Institutional Review Board. The survey was conducted in accordance with the relevant guidelines and regulations and an informed consent was obtained from all the participants.

## Results

The demographics of the participants are provided in Table 3. The majority of participants were female (66%), 34 to 54 years of age, (70%), and worked as CHVs for more than 5 years (76%). As CHV is a volunteered position, two-thirds indicated they were employed. In the month-long observation period immediately after the training, CHV evaluated 1,443 individuals. Of these, 154 were referred and received at the PC clinic and an additional 58 home visits were conducted jointly by the CHV and a PC provider. Telephone conversations were also conducted with CHVs making 110 calls to a patient and 129 calls to a PC provider. One year follow-up assessment was conducted between March 2022 and May 2022, with 79 CHVs returning to participate in a face-to-face knowledge assessment and FGD.

**Table 3:** Socio-demographics

Age group	Male	Female	Total
< 35	3	6	9 (11.39%)
35–44	7	17	24 (30.38%)
45–54	11	20	31 (39.24%)
> 55	6	9	15 (18.99%)
Total	27 (34.18%)	52 (65.82%)	79

The impact assessment was divided into 4 Levels which were assessed at the time of training and at the one-year follow-up.

### Level 1 Reaction

**Fig. 1:**
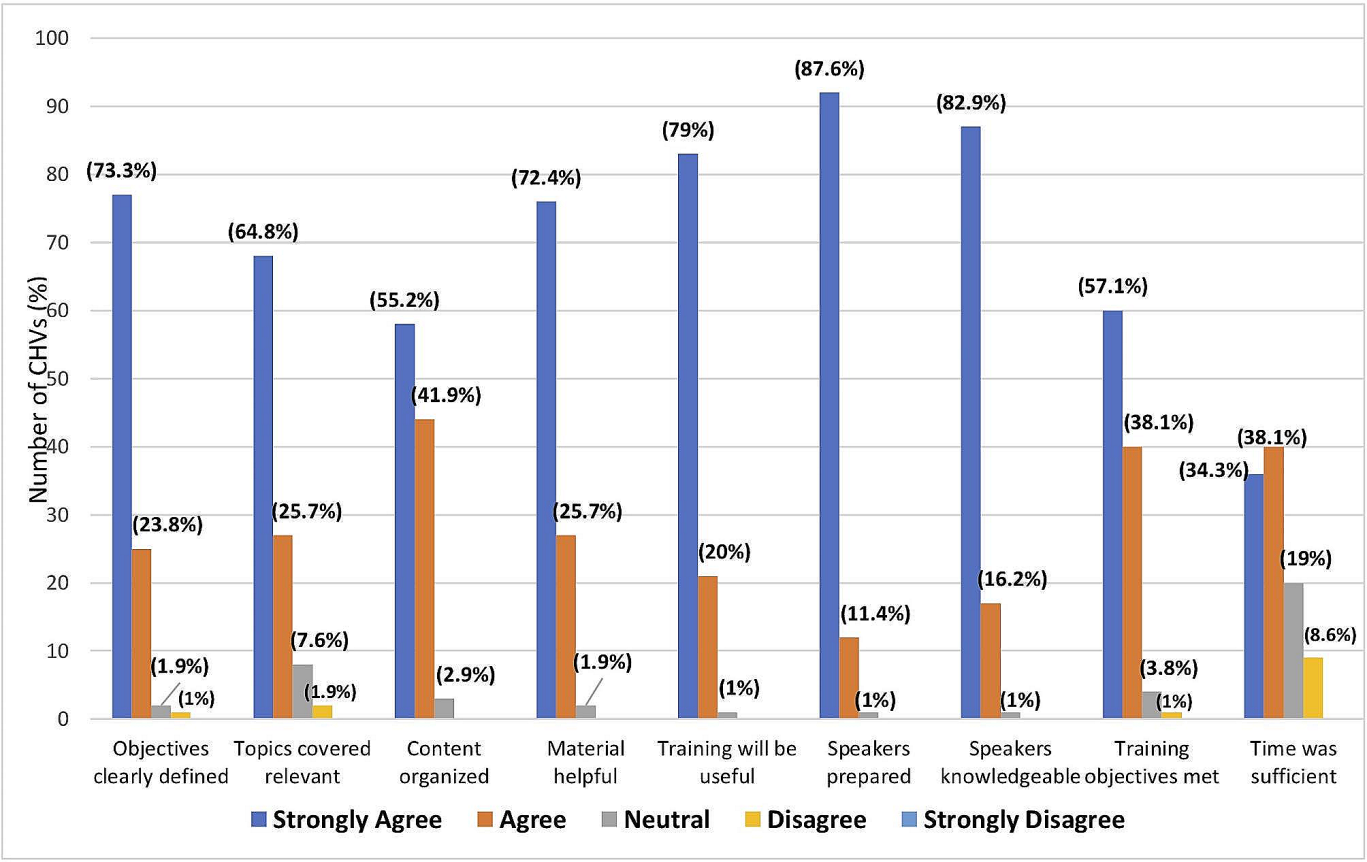
Training evaluation. *Abbreviations* CHVs - Community health volunteers

This was assessed at the time of initial training (see Fig. 1). Most of the 105 participants agreed or strongly agreed that the training was relevant (90.8%), will be useful to them in their work (99%), and the training materials were helpful (98%). Approximately two third (70%) of the participants agreed or strongly agreed that the duration of the training was sufficient.

### Level 2 Learning

Table 4 shows the levels of self-assessed knowledge on various domains of PC including physical symptom assessment, spiritual issue assessment, provision of basic nursing care, and bereavement care. There was an overall statistically significant improvement in all the domains when comparing pre- and post-training values.

**Table 4:** Self– assessed level of knowledge pre–and post–training

Knowledge aspect	Pre-Training knowledge level	Post- Training knowledge level	Improvement	p value
COVID 19	2.6	4.7	2.1 (77.9%)	*p* < 0.001
Pain assessment	2.6	4.5	1.9 (75.9%)	*p* < 0.001
Assessment of other physical symptoms	2.7	4.5	1.8 (68.9%)	*p* < 0.001
Application of non-medical interventions	2.5	4.3	1.8 (75.3%)	*p* < 0.001
Prevention and care of bedsores	2.5	4.5	2 (79.9%)	*p* < 0.001
Feeding a disabled or bedbound patient	2.5	4.6	2.1 (82.7%)	*p* < 0.001
Turning a disabled or bedbound patient on bed	2.3	4.5	2.2 (92.9%)	*p* < 0.001
Cleaning and dressing of wounds	2.4	4.5	2.1 (86.5%)	*p* < 0.001
Stoma care	2.1	4.1	2 (95.1%)	*p* < 0.001
Spiritual issues assessment	2.8	4.6	1.8 (66.2%)	*p* < 0.001
How to provide grief and bereavement care	2.3	4.5	2.1 (91.3%)	*p* < 0.001

p value from two–tail paired t–test

Table 5 shows self-assessed confidence levels on pain assessment, change of urine bag, prevention and care of bedsores, turning a patient in bed, feeding procedures, patient referral, spiritual assessment, communication skills, ethical principles pre–and post–training. Statistically significant Improved confidence was reported in all domains except for patient referral.

**Table 5:** Self– assessed level of confidence pre–and post–training

Care skill	Pre-training confidence level	Post-training confidence level	Improvement	p value
Pain Assessment	3.74	4.58	1.32 (35.40%)	*p* < 0.001
Change Urine Bag	3.11	4.35	1.58 (50.65%)	*p* < 0.001
Bedsore Care	3.50	4.29	1.15 (32.90%)	*p* < 0.001
Turn Patient in Bed	3.82	4.54	1.03 (26.99%)	*p* < 0.001
Feeding Procedures	3.49	4.31	1.23 (35.16%)	*p* < 0.001
Patient Referral	4.38	4.64	0.56 (12.70%)	0.065
Spiritual Assessment	3.90	4.28	0.80 (20.50%)	0.013
Communication Skills	3.93	4.33	0.75 (19.08%)	0.003
Ethical Principles	3.51	4.15	1.00 (28.46%)	*p* < 0.001

p value from two–tail paired t–test

### Level 3 behavior

**Fig. 2:**
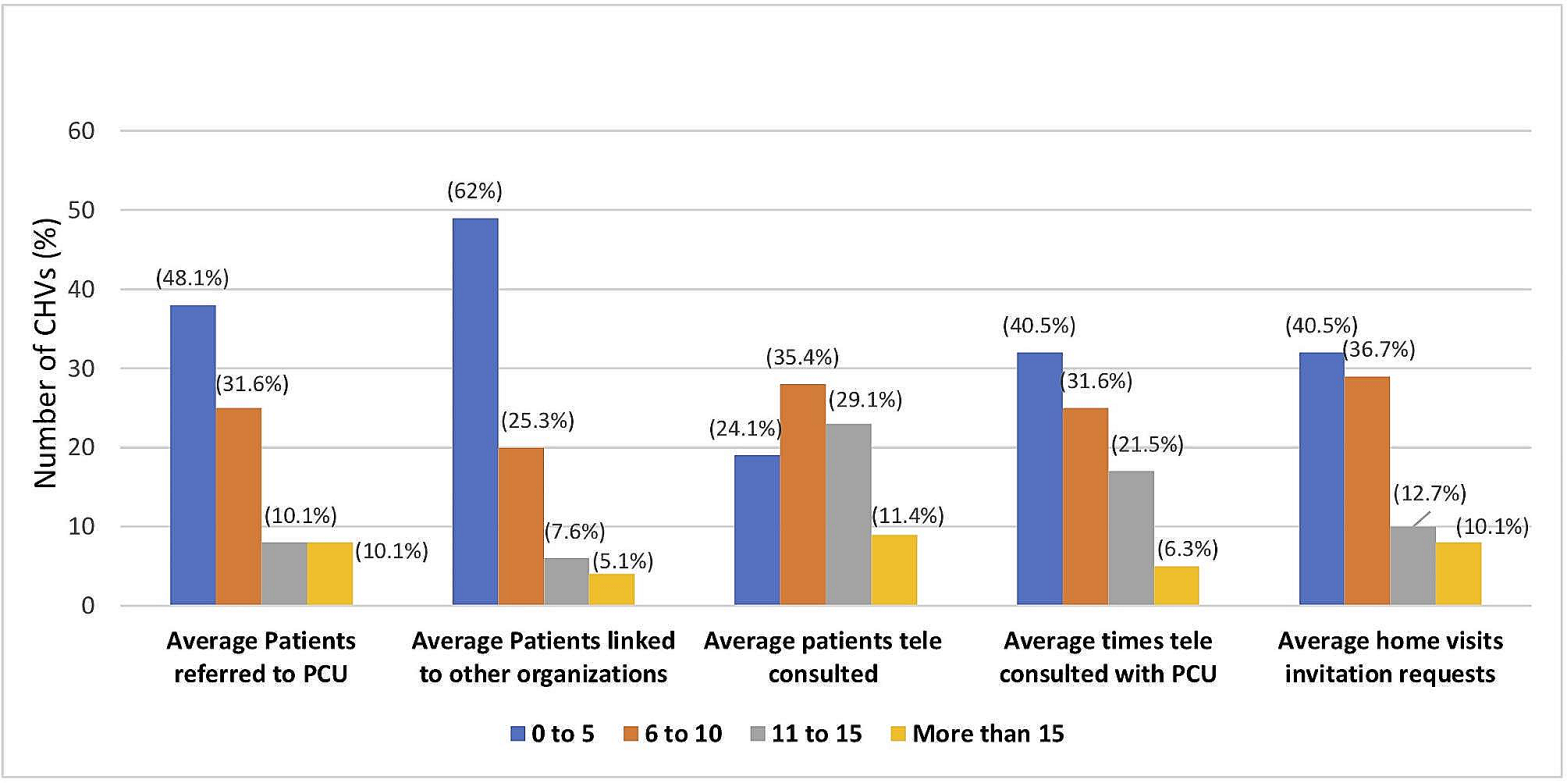
Some of the activities performed by CHVs during the first 4 weeks after the training. *Abbreviations* PCU – palliative care unit, CHVs – community health volunteers

The average number of assessments performed during the month after training varied with a third of CHVs assessed ≤ 10, approximately half assessed between 11 and 20, and the remaining assessing over 20 community members. Figure 2 shows the average number of selected activities performed first month after training. During this period, approximately 6 or more patients were referred to PC clinics, linked to other organizations and tele–consulted with CHV by approximately 50%, 40% and 76% of the CHVs respectively. In addition, approximately more than two third CHV tele consulted with PCU 6 or more times and more than half invited a PC provider to accompany them on a home visit.

Level 4 Results: During the training a copy of the training manual was given to all the CHVs. Two thirds of the CHV’s were using the training manual either most or all the time during their community visits. Approximately 90% of the participants reported that they saw the relevance of the training most or all the time during their community visits. Approximately 90% CHVs reported that they used the assessment tool while assessing community members most or all the time and 99% felt the assessment tool was helpful to assess the condition of the community member. When asked about the ease of use of the assessment tool, only 6% found it difficult to use.

All of the CHVs reported an increase in the number of consultations from the community and 94% reporting that they facilitated community education on PC. Part of the training program was to enable participants to train other CHVs in their community units. 97% felt they could utilize the training manual to train others and 97% reporting they trained other CHVs and 94% trained members of their community.

In addition to the quantitative assessments, 79 CHV returned one year after the initial training to participate in a semi-structured interview in which six questions were used to obtain feedback. The major themes that arouse from the FGD are listed in Table 6 and details are available in Table 1, supplemental data. Attentive listening and counseling were improved and led to open discussion of illness, confidence in addressing misinformation, better symptom assessment and medication adherence interventions. The training also improved caregiver interactions and spiritual care. The CHV felt empowered to make referrals to PC providers, encourage patients to use technology in their care, and educate family on basic care issues for ill patients. The communication skills were noted to improve interpersonal relationship with patients and family and helped the CHV deal with the stress of caring for patients at the end of life.

**Table 6:** Major themes from FGDs

Please describe to me how it has been for you to manage patient medication adherence?
• Engaging with and training caregivers
• Counseling on advantage of medication
• Offered counseling around spiritual issues that conflict with medication adherence (ex. witchcraft as cause of disease)
• Frequent home visits
• Alerted other healthcare providers when adherence is in question
• Developed a network of supporters for medication adherence (CHV, Neighbor, Caregiver, Doctor)
**Please describe to me what changes have occurred in your patient care practice since you have completed this training?**
• Incorporated counseling of patients and caregivers; counteracting misinformation address patients who feel they are a burden or have feelings of hopelessness
• Counseling led to improved CHV-patient relationship with more open discussion of illness by patient
• Instructed patients on use of technology to improve self-care (clock and phone alarms for medication reminders)
• Improved social life: counsel patients on use of technology to decrease social isolation
• Improved personal hygiene habits along with education of patients and caregivers
• Counseling on benefits of insurance coverage
**What part of the training was new to you?**
• Interpreting symptoms
• Improved communication skills with patients and family
• Dressing changes, catheter care, bedsore prevention, massage
• Early cancer treatment (? cure) is possible
• The importance of spiritual care, engaging community spiritual leaders in patient care
• Making referrals to nearest healthcare facility
• COVID-19 transmission and prevention
**How did the training change your practice?**
• Improved attentive listening and patients
• Improved interpersonal relationships with patients
• Offering support in accessing healthcare
• Recommended cost-effective alternatives to current interventions
• Embraced support networks in the community
**How did you cope while seeing the very sick patients?**
• Training improved understanding of palliative care
• Apply the new skills helped CHV develop courage and hope
• CHV grew closer to the patient and family based on the new skills
• Encouraged patient interactions with local spiritual leaders
• Have the support of palliative care doctors and nurses helped CHV cope with ill patients
**How do you think the community benefits from your training?**
• Patient attitude change from viewing sickness as witchcraft
• Exhibiting new skills changed the view of CHVs, some community members began to refer to CHV as “doctor”
• Improved community trust and confidentiality
• training of caregivers allowed them to help a family member and others in the community
• Increased awareness that caring can be done in the community, not just the hospital
• Engaged the community in assisting patients and families

## Discussion

The WHO and other organizations have encouraged CHV involvement in palliative care delivery. In 2020, MacRae et al. addressed this issue in LMIC and found only 13 studies appropriate for their review [10]. For a more recent evaluation, we performed an OVID search (https://www.wolterskluwer.com/en/solutions/ovid) on February 14, 2024 using the keywords Community Health Workers (CHW), CHV, and Palliative Care and found 27 references. Searching CHW, CHV and Hospice did not add any additional references. The majority of publications were from high income countries where CHV expressed a positive attitude towards end-of-life care and including CHV in PC delivery may decrease medical costs [14, 15]. CHV were successful in to promoting advance care planning, utilization of hospice and palliative medicine, particularly in underserved populations [16–24]. Culturally based care was also an important factor in improving PC outcomes [25]. Most of the CHV in high income countries were supplementing an existing palliative care workforce. In contrast, CHV may be the only resource available for home-based care in LMIC and there are few publications addressing their role in palliative care delivery. Over a decade ago, Uganda developed national policies that included adding palliative care to the scope of CHV activities and other countries have followed suit [26] (2). A national PC policy in South Africa promotes CHV involvement but a report in 2022 recommended a needs assessment [27]. The study also noted CHV expressed uncertainty regarding their role in home-based palliative care. In general, publications recognize that adding PC to CHV practice represents a change from their current focus on preventative and general primary care needs. While publications have highlighted the need for research studies [10, 12, 27], our report is one of the few addressing CHV PC training and implementation in the LMIC setting.

The training curriculum described here was designed to cover the three main domains of PC which include physical symptom assessment and treatment, spiritual and social care and grief and bereavement care. Special emphasis was placed on the communication skills used in discussing difficult topics. The manuscript present pre- and post-assessments of training. CHV found the training of value, increased referrals to PC providers, and empowered the CHVs to alter their practice by providing a variety of PC services.

CHVs live within the community they serve and have been selected by the members of the same community, speak the same language and are from the same cultural background which is an added advantage in performing their roles [8]. In Kenya, their key roles and responsibilities include making home visits to assess health situations, share health improvement and prevention information from the MOH, treat common minor illnesses and injuries, and address maternal, newborn health and childhood health issues [8, 9]. The study found that with additional training and linkage to palliative care providers, CHVs can identify the PC needs of their community and appropriately act within their scope of practice. This includes a role in providing psychosocial and spiritual support as well as newly learned clinical care skill.

Following the training the CHVs used the training manuals and assessment forms provided to them during their community visits. We believe that these documents provided a stepwise guide to community member assessment and intervention and positively impacted the knowledge and confidence to initiate telephone consultations with PC mentors.

CHVs reported that through the training they have learnt new skills and the importance of biopsychosocial nature of chronic illnesses which they incorporated into their practice. They also appropriately identified patients for referral. At one year follow up, the CHVs also reported an increase in the number of PC consults they received from community members. In the FGD, several CHVs commented that by applying their new skills the respect shown to them by the community improved. The CHVs recognized the benefit of tapping into the existing community resources to help the sick community members. The CHVs trained caregivers who were then able to care for their sick family member and others in the community. This is consistent with the study by Soderhamn et al. showing a network of volunteers (beyond CHVs) can address patient needs and mobilized resources in the community [14].

This survey has shown a statistically significant overall improvement in knowledge and confidence in conducting basic nursing procedures like cleaning and dressing of wounds, stoma care, change of urine bag and feeding procedures except for patient referral. We believe that this can reduce unnecessary hospital visits especially for bedbound patients and reduce transport and procedure costs, but the clinical and financial impact will require further study. Greater access to home PC has the potential to allow patients with no access to home hospice to remain at home as they near end-of-life. A population-based study done in Kenya reported that approximately 50% favored dying at home [15]. Interestingly, 23.7% stated dying at home was the least preferred place, which some credited to the low availability of resources. This suggests an unmet care needs that CHVs could fulfill [15].

There were a number of limitations to our study. CHVs are familiar with patient referral as this is part of their monthly reporting requirements. They rated this skill the highest in terms of pre training confidence. This could explain the reason patient referral was not statistically significant and suggests this part of the training curriculum could be revised in future trainings. CHVs embraced teleconsultation which was facilitated by the robust telecommunication infrastructure in Kenya where the number of subscribers per capita is higher than most countries, including the United States (https://data.worldbank.org/indicator/IT.CEL.SETS.P2). The provision of PC by CHV may be less effective in countries will limited telecommunication coverage. Our training also empowered CHVs to train other CHVs but the impact of the peer training on patient care is unknown. CHV also were within counties that had an active palliative care clinic that was part of a larger hub and spoke model of PC support, areas where access to PC providers is limited may decrease the effectiveness of training. The availability of a 24-hour PC hotline and the small amount of financial support for phone fees are also components that could impact replication of our findings. We also had cooperation from the MOH in allowing CHV to participate in the training, which was likely influence by the Kenya Palliative Care Policy 2021–2030 which sets goals for increasing access to PC. Sustainability will require continued support by the MOH in allowing CHVs to incorporate PC into their other duties. Adding palliative care services to the CHV skill set may cause a negative impact on performing existing responsibilities as an unintended consequence. While this was not directly assessed in questionnaires, CHVs did not mention this as a limitation during semi-structured interviews.

## Conclusion and recommendations

PC specific training which includes practicum, provision of support material and mentorship is relevant and it improves skills, knowledge, and confidence amongst CHVs which changes their practice and behavior. This change has been reported by the CHVs to benefit the community. The components of this training can prepare CHVs on PC task shifting in the community. In addition, the training led to increase referrals to palliative care providers. Our study suggests CHVs can play an important role in a hub and spoke model of PC provision in resource limited settings. Further studies are warranted to assess the impact of the CHV activities from the community perspective, reduction in unnecessary hospital visits, reduced costs and also to review the role of CHVs in performing specific activities like increasing access to morphine for pain management.

### Electronic supplementary material

Below is the link to the electronic supplementary material.

Supplementary Material 1

Supplementary Material 2

## Data Availability

The datasets used and/or analyzed during the current study are available from the corresponding author on reasonable request.
