# Supplementary material for: Impact of training on knowledge, confidence and attitude amongst community health volunteers in the provision of community-based palliative care in rural Kenya
Source: BMC Palliat Care. 2024 Apr 11;23:97. doi: 10.1186/s12904-024-01415-5 (PMC11007868; doi:10.1186/s12904-024-01415-5)
Supplement: Supplementary file 1 — Supplementary Material 1 [file 12904_2024_1415_MOESM1_ESM.pdf]

SUPPLEMENTAL DATA

A series of 10 semi-structed Focus Group Discussion were held with a total of 79 Community Health Volunteers. Six questions were posed to the group. The sessions were recorded and anonymized responses were reviewed and categorized by a study author (EK) and summarized below along with selected quotes that illustrate the major themes that arose during the discussion. Note: healthcare workers are proficient in the two official languages of Kenya, Swahili and English and often utilize both in conversations. The quote is provided in italics, and when a translation is required it is provided within the parentheses following the quote.

QUESTION 1. Medication adherence – The question posed to participants was, "Please describe to me how it has been for you to manage patient medication adherence?

The CHVs managed patient medication adherence with a focus on the caregivers. This involved frequent home visits along with identifying the challenges to adherence. CHV gave advice on the importance of taking the medication on time and as prescribed. They also provided health education and collaboratively worked with the caregivers to help them manage the patient's medication. CHV quotes that illustrate themes noted in the focus groups are provided below.

Regarding frequent home visits:

- "*wengine walifaidika na regular visits sasa wanaona kweli tuko na mtu mwenye anantutemebela kila siku na anpeleka mtu wetu kwa hospitali"* (regular visits that are encouraging to community members who feel appreciated, supported and the fact that they are taken to hospital)

On ensuring that the correct dose of medication is taken, CHV helped place medications in separate containers.

- "*Okay another one is to teach the caregiver how to differentiate types of medicine you just put others in another packet to show a difference ndio wasimix dawa wajue gani sio gani ni gan*i." (so they do not mix medication; they can differentiate the medication given).

CHVs monitored medication adherence in follow-up visits by performing tablet counts, confirming the medicine schedule and confirming that the medicines are administered the medication accordingly. CHVs also alerted health care providers when adherence was in question.

- "*You report in case hakuna hiyo adherence you report to the facility penye anachukua dawa ili waone the next action itakuwa ni gani"* (reporting to the facility where the patient gets the medication from, so they can advise on the next cause of action)
- *" I found a patient who had never been to hospital since so I talked to her neighbor then I got her caregiver. I discussed with her then the patient came to hospital then the doctor talked to him they to take the drugs and they went home. I think she is now doing right".*

For patients who refused to take medicine because of spiritual beliefs, the CHVs would use the training in spiritual care to counsel patients on the medical basis of their illness.

- *"some of the patients tend to believe that they had been bewitched and so we have changed them to know that they are sick because sickness is also there not witchcraft"*

Viewed as an innovation, the CHVs created a collaborative chain of supporters, each taking their own roles in supporting a patients' medicine adherence. This chain is summarized as: CHV —-> Neighbor——>Caregiver —-> Doctor—-> Medication taking. Q

QUESTION 2. Changes in CHVs Practice The question posed to participants was, "Please describe to me what changes have occurred in your patient care practice since you have completed this training?"

CHVs taught patients and caregivers how to use technology in care management. Examples for using phones or clocks were:

- ”*I took the contact of my patients so if it is the date to take medicine I ring the caregiver to ask if they have got medicine or not".*
- *"I was able to educate the caregivers to know how to use the clock and some of them were using the alarm on the phone to make sure that when time reaches for the patient to take their drugs the alarm is on and they are given their drugs on time".*

CHVs reported new hygiene habits which they believed reduced infections. Both CHVs and individuals in the community had changed hygiene practices.

- *"sometimes wacha niseme nilikuwa labda mchafu yaani nilibadilika wakati nilikuwa naenda kwa mgonjwa ninakuwa mchafu sasa hapa inafanya nikakuwa massif"* (let me say that sometimes I was not clean, but this changed when I started visiting patients that I had to embrace cleanliness)

Improved quality of life was noted as CHVs incorporated new counseling skill into their practice. Counseling reduced stigma for patients encourage them to go back to socializing. This also led to community efforts to identify patients with hidden ailments who were in need of support. CHV commented on rejuvenating a love for life and bringing back hope to patients who had lost it.

"another thing is that there are relatives people wameaccept kuna rejection yaani kukataliwa llakini kuna improvement hawakataliwi kwa maana tumejaribu kuongea na relatives wao kujua maana ya kuchunga mtu wao akiwa mgonjwa na wengi hata wamepata kutemembea yaani hawaogopi tena kutemebea nje wameanza sasa hiyo relashipship mzuri hakuna hiyo stigma tena" " (another thing is that there are relatives, people who have accepted that rejection exists, but there is improvement because they are not rejected because we CHVs, have spoken with their relatives concerning caregiving to a family member who is ill, and majority are now able to go out and meet other people without being stigmatized)

Bringing support for use of urinary catheters resulted in improved social networks for patients, allowing them to once again link up with friends for social gatherings thereby ending loneliness. CHV reported an attitude change as patients no longer saw themselves as a burden to their family. CHVs also reported their attitude changed to a more positive one.

CHV also now counseled patients with trouble paying for care and medications on the potential advantages of enrollment in the national health insurance plan (NHIF) and believed this led to an increase in insurance enrollment and hospital patronage.

- "…….*patients wetu walifaidika kwa sababu wale hawakukuwa na NHIF waliweza kupata NHIF"* (those who had no NHIF they were being assisted to enroll and get one)

Additionally, now that CHVs were counseling patients and counteracting misinformation they found caregivers were giving information freely, which was not the case before. With assurance

of confidentiality, patients now felt free to explain their condition. In turn, this improved the ability of patients to explain their disease and symptoms with more accuracy. This aided the CHVs as they offered advice for treatment. Finally, counseling led to increase in knowledge; especially for HIV mothers regarding childcare, socializing opportunities/networks, hygiene, and balanced diet.

QUESTION 3: New information skills: CHV were asked "What part of the training was new to you?"

Speaking on care skills, the CHVs noted that they were able to interpret symptoms,

- "*baada ya training sasa naweza tazama mgonjwa physically hivi alafu najua shida yake ni nini*" (After the training, I can tell by looking at the patient physically what the problem is)

Others indicated that they were able to read into the patients feelings and identify what the issue was:

- "now I can know or identify or read the feelings of my patients,"
- "during my house to house visitation I was able to assess the amount of pain the patient had and refer if it was worse."

CHVs noted a new understanding of how to communicate with patients, how to deal with hostile families and environments, and how to develop friendly relationships.

- "*in addition, after the training I leant how to create that communication atmosphere with the patient to create the rapport to counsel"*

CHV listed the following skills that were new or improved: wound dressing changes, catheter care and changing urine bags, bedsore prevention, performing massage, turning a bed ridden patient, body therapy, home therapy, exercise, handling a patient for exercises outside the house, how to move one from the house to the outside, assisting a patient who is vomiting, feeding a patient who is unable to feed himself/herself, care of feeding tubes, changing bed linens and repositioning a patient while minimizing pain. Wound care was a new skill that was of significant addition to CHV work:

- *"mbeleni hata tulikuwa hatutajua vile ya kudress mgonjwa kidonda lakini saa hizi tumejua sababu tulikuja hapa tukafunzwa tukasoma vile ya kudress mtu mwenye ako na kidonda sas atufanya tukijua",* (prior before training, we knew nothing about dressing wounds, but now we know)
- *"After training wagonjwa wangu watatu saa hii wameimprove wanaweza kutemeba nimejua kuwafunga kidonda sasa kinaendelea kinapona sasa wameniweka jina mama palliative"* (After the training, my three patients improved, they can walk, I know how to dress wounds, which are healing and now the patients have a name for me, Mother Palliative)

The CHV had an increased understanding that early cancer treatment was possible, and informing patients about this was embraced by the CHVs. The CHVs also encouraged patient interactions with local spiritual leaders, who offered prayer and whose physical presence provided spiritual comfort.

The CHVs also learned about spiritual care and addressed it in their patient interactions.

- "we *encouraged the patient that God has the ability to heal, and not only by using medication, is a person made well, but also reliance on God is key".*

Another care practice change was making referrals to nearby healthcare facilities since they now knew who to call.

- "we were able to do refer as many patients as we can, *mara nyingi tukipata wagonjwa katika kijiji chute kitu cha kwanza tunaona kama anahitaji referral tunapea…"* (we do so whenever we see the need).

However, the CHVs were quick to note that some villages where resistant to accepting referrals outside the community. Interestingly, they also confessed that their adherence to COVID-19 preventative practices (handwashing, sanitizing, and social distancing) improved as some communities they visited were actively practicing the guidelines.

QUESTION 4: Practice Changes: The CHV were asked, "How did the training change your practice?"

Training led to improved attentive listening and patience that resulted in better interpersonal relationships which enabled CHVs to better manage their patients.

"*change ingine how tulikuwa tunacommunicate na hao tunapigiana simu tunajuliana hali na mgonjwa sasa mgonjwa aliulalaje anasikiaje sasa tukacommunicate na the caregiver ama kama mgonjwa anawezakuongea nawezakuonge na yeye through the telephone*" (another change is that we communicated with the caregiver to know the state of the patient, or if it was possible we spoke directly to the patient by phone)

- "….*it changed my home visits because it made me know that whenever I visit a patient frequently is when they open up they can tell me whatever they could not tell me if I visit them once a month*"
- *"… after training I can now sit and listen ….. after the training I know how to communicate with the patient I must wear his/her shoes"*

The training also led CHV to offer support when patients needed to access healthcare, as illustrated in the two examples below.

- "*ingine pia katika office yetu tuko na means of transport kama hizi ambulance kufanya organization jinsi ya kufika hospitali"* (within our office we have the means of transport like ambulances enabling us to organize for ways of getting to the hospital),
- "… *after getting this training palliative I was exposed to the facility and the community whereby I had that empowerment to reach to the facility whereby my client/patient would not be able to reach there and I created that link for further treatment*".

The CHVs shared their struggles and challenges on scheduling visits and transportation, with patients changing schedules and balancing this with the size of their catchment area which might involve 12 different villages.

After the training the CHVs were able to recommend cost effective options when making care decisions:

- *"so form the training I realized that we can use local foods to improve the nutrition of our patients".*

- *“there is another community member who benefited by saving because he was supposed to travel to a nearby facility just to replace the catheter so I was able just to help them to change”*

CHVs embraced other support networks in the community including counseling services and referring patients to nurses, and also linking patients to sponsors from the political sector and/or non-governmental organizations.

- “....*also before the training just maybe knew that the patient can just get some support or self-reliance but after the training came to know that we can also link them in different facilities and sponsors like MCA’s and whatever like non-governmental NGO’s to get in support”*

QUESTION 5: Care of palliative patients: The CHV were asked, “How did you cope while seeing the very sick patients?”

The CHVs acknowledged that they better understood what palliative care entailed after the training.

- *“PC was just new to me I had not known anything to do with PC I was just hearing PC PC, after the training I knew everything about PC”.*
- *“the whole PC was very new to me and I could not captured up with diagnostic terminologies but to some level I have now the information”.*
- “*I feel courageous and those people have become social and identified me as one of the helpers in the community”*
- “*I could look for a way if I realize that maybe this patient might die at any time I look for a way talk to the family members to prepare them psychologically and also to encourage them and the patient himself/herself so that they can be stable while taking care of that patient”,*

The CHVs found the link to doctors, nurses and others at health facilities helped cope with very sick cases,

- *“also I would first make sure I do some a follow up confirmations to know the cause of the seriousness situation to which the patient is undergoing because the patient might be in that situation because she/he has not been taking medicine or maybe she/he needs a referral for some further treatment”*

QUESTION 6: Community Benefits: Participant were asked, “How do you think the community benefits from your training?”

Several community benefits were noted. The first being an attitude change from viewing sickness as witchcraft,

- “*kwangu mi naona community imebenefit kuna wagonjwa walikuwa wamekaa tu wameamini uchawi hawakuwa wanapeleka mtu kwa hospitali sasa imesaidia wanapeleka wagonjwa hospitals”* (attitude change from believing that sickness is as a result of witchcraft, after seeking for healthcare and responding well to medication),

Secondly, the application of new skills changed the communities view of CHVs as being an individual that can play an important role in local healthcare.

- “..*ya mwisho pengine walikuwa wantuoan tu hivi lakini after training we are somebody”* (that the CHV was seen as a normal person but not after the training where they are recognized and respected)”

- *"community see me as their savior kwa sababu ukishafika huko wanajua tu watapona" (*that her presence is a guarantee to healing*)*
- *"now they recognize that these people CHVs are the right people to be consulted whenever anything that is near which has caused a problem they can help or transfer and are able to refer anybody who is sick",*
- "*pia mimi nilipata kujua ya kwamba kumbe hata mimi ni daktari kwa sababu kama ninawezamwekea hiyo catheter nibadilishe urine bag nikajioana kumbe hata mimi pia nimekuwa daktari mwingine"* (I got to know too that I am a doctor because I can change a catheter , seeing myself as another doctor)

"*despite visiting my patients who are In the interior I used just sacrifice and reach them on a it was during a rainy season and the roads were muddy but having that passion now to serve the community I was used just able to go through the muddy roads and just serve them and for doing that they have gained a lot of confidence and trust in me, ….the community has also recognized that they find CHV very important because they have seen the confidentiality that we have kept because when we go to patient you keep their secret"*

Training of caregivers who could extend care from family member to others in the community also benefited the community. The CHVs discussed educating community members in rendering service to the patients, including getting patients to health facilities, good hygiene practice, and mobilizing adults and children to provide care. Families also benefited from referrals to other patient support programs. Caregivers within the community benefited CHV training on patient care, and this in turn had a rippling effect on the community, who also learned from caregivers through family and friends:

- *"according to me there was a child who used not to walk but I went there I trained caregiver how to do practice to the child now she used to do practice to the child to know how walk now the child is walking without support form caregiver"*

CHV activities and training of families and community members showed that care to patients could be done in the absence of the CHV. This led to an increased awareness that care can be done in the community, not just the hospital. This was a cost saving benefit to the community.

- *"something new ni kwamba kuna patients pia wenywe wako nyumabani I thought palliative care unawezafanyia tu kwa hospice hata nyumbani pia tunaweza kumhudumia vile ianhitajika"* (something new is that we have patients who are home, but I thought palliative care can only be done at the hospice, but now even at home a patient can be cared for the way it is required)

"something new ni kwamba kuna patients pia wenywe wako nyumabani I thought PC unawezafanyia tu kwa hospice hata nyumbani pia tunaweza kumhudumia vile ianhitajika"

Finally, families in need were recognized and supported by CHVs and the community as well. For example, families with no resources and needed help were supported financially and/or in kind by the community. One example of community support:

- *"wale walihitaji wheelchair waliweza kupata"* (they asked for a wheelchair, and they got it)
