# Supplementary material for: Impact of training on knowledge, confidence and attitude amongst community health volunteers in the provision of community-based palliative care in rural Kenya
Source: BMC Palliat Care. 2024 Apr 11;23:97. doi: 10.1186/s12904-024-01415-5 (PMC11007868; doi:10.1186/s12904-024-01415-5)
Supplement: Supplementary file 2 — Supplementary Material 2 [file 12904_2024_1415_MOESM2_ESM.docx]

**SUPPLEMENTARY FILE 1: QUESTIONNAIRES**

**Evaluation tool used post training *(administered immediately post 3 day training)***

| Question | Strongly agree | Agree | Neutral | Disagree | Strongly disagree |
| --- | --- | --- | --- | --- | --- |
| The objectives of the sessions were clearly defined |  |  |  |  |  |
| The topics covered were relevant to me |  |  |  |  |  |
| The content was organized and to follow |  |  |  |  |  |
| The material distributed were helpful |  |  |  |  |  |
| This training experience will be useful in my work |  |  |  |  |  |
| The speaker[s] were well prepared |  |  |  |  |  |
| The speaker[s] were knowledgeable about the topics |  |  |  |  |  |
| The training objectives were met |  |  |  |  |  |
| The time allocated for the training was sufficient |  |  |  |  |  |

What did you like the most about this training?

What aspect of the training could be improved?

Which area mostly understood?

Which area needs further training?

General grading in palliative care.

**Confidence rating scale *(administered pre and immediately post 3 day training)***

1. I can assess a palliative care patients’ pain

- Pre training 1(strongly not confident) 2(not confident) 3(neutral) 4(confident) 5(very confident)
- Post training 1(strongly not confident) 2(not confident) 3(neutral) 4(confident) 5(very confident)

1. I know how I can change a urine bag for a palliative patient o catheterization

- Pre training 1(strongly not confident) 2(not confident) 3(neutral) 4(confident) 5(very confident)
- Post training 1(strongly not confident) 2(not confident) 3(neutral) 4(confident) 5(very confident)

1. I know how to take care of a patient with bedsore

- Pre training 1(strongly not confident) 2(not confident) 3(neutral) 4(confident) 5(very confident)
- Post training 1(strongly not confident) 2(not confident) 3(neutral) 4(confident) 5(very confident)

1. I know how to turn a patient on the bed

- Pre training 1(strongly not confident) 2(not confident) 3(neutral) 4(confident) 5(very confident)
- Post training 1(strongly not confident) 2(not confident) 3(neutral) 4(confident) 5(very confident)

1. I know feeding procedures for a patient who needs palliative care

- Pre training 1(strongly not confident) 2(not confident) 3(neutral) 4(confident) 5(very confident)
- Post training 1(strongly not confident) 2(not confident) 3(neutral) 4(confident) 5(very confident)

1. I know how to do patient referral to health facilities

- Pre training 1(strongly not confident) 2(not confident) 3(neutral) 4(confident) 5(very confident)
- Post training 1(strongly not confident) 2(not confident) 3(neutral) 4(confident) 5(very confident)

1. I know how to assess spiritual needs of a patient

- Pre training 1(strongly not confident) 2(not confident) 3(neutral) 4(confident) 5(very confident)
- Post training 1(strongly not confident) 2(not confident) 3(neutral) 4(confident) 5(very confident)

1. I feel well equipped with effective communication skills in palliative care

- Pre training 1(strongly not confident) 2(not confident) 3(neutral) 4(confident) 5(very confident)
- Post training 1(strongly not confident) 2(not confident) 3(neutral) 4(confident) 5(very confident)

1. I feel able to offer palliative care with regard to human ethical principles.

- Pre training 1(strongly not confident) 2(not confident) 3(neutral) 4(confident) 5(very confident)
- Post training 1(strongly not confident) 2(not confident) 3(neutral) 4(confident) 5(very confident)

**CHV survey for community based palliative care training: *(Administered 12 months after the training)***

1. What is your age
   1. <35 years of age
   2. 35-44 years of age
   3. 45-54 years of age
   4. >55 years of age
2. What is your gender
   1. Male
   2. Female
3. Duration you have been a Community health Volunteer
4. less than 6 months
5. more than 6 months but less than 1 year
6. more than 1 year but less than 3 years
7. more than 3 years but less than 5 years
8. more than 5 years
9. Highest level of education attained
   1. primary level
   2. secondary level
   3. university/college level
   4. have not been to school
10. employment
    1. self employed
    2. employed
    3. not employed
11. How many patients on an average did you assess during the first 4 weeks after the face-to-face training?
    1. 0-10
    2. 11-20
    3. 21-30
    4. >30
12. How many patients on an average did you refer to the PCU or other facilities for further care during the first 4 weeks after the face-to-face training?
    1. 0-5
    2. 6-10
    3. 11-15
    4. >15
13. How many patients on an average did you link to other organizations or resources to receive special assistance (e.g., wheelchair, financial support, food, transport, medicines and etc.) and continuous care?
    1. 0-5
    2. 6-10
    3. 11-5
    4. >15
14. How many patients on an average tele consulted you during the first 4 weeks after the face-to-face training?
    1. 0-5
    2. 6-10
    3. 11-15
    4. >15
15. How many times on an average did you tele consult with the PCU during the first 4 weeks after the face-to-face training?
    1. 0-5
    2. 6-10
    3. 11-15
    4. >15
16. How many invitations requests for home visits to the PCU did you make on an average during the first 4 weeks after the face-to-face training?
    1. 0-5
    2. 6-10
    3. 11-15
    4. >15
17. Have you used the training manual during your routine community service?
    1. Not at all
    2. Sometimes
    3. Most of the times
    4. All the times
18. Has the training been relevant to you when you perform your community service?
    1. Not at all
    2. Sometimes
    3. Most of the times
    4. All the times
19. a. How frequently did you use the patient assessment form to help you assess the patients?
    1. No not at all
    2. Sometimes
    3. Most of the times
    4. All the times
20. Is the patient assessment form helpful to you in understanding the condition of the patient?
21. YES
22. NO

If NO, please explain why?

______________________________________________________________________________________________________________________________________________________________________________________________________

1. Do you find it difficult to use and fill the assessment form?
2. YES
3. NO

If YES, please explain why?

_______________________________________________________________________________________________________________________________________________________________________________________________________________

1. How often are you able to fill the assessment form independently without any help from your colleagues or other healthcare providers?
   1. No not at all
   2. Sometimes
   3. Most of the times
   4. All the times
2. Do you think using the training manual together with the experience and knowledge you have gained you can train other CHVs?
   1. YES
   2. NO
   3. If NO, please explain why

________________________________________________________

________________________________________________________

________________________________________________________

- 1. If YES, how many other CHVs have you trained?
  2. 0-5
  3. 6-10
  4. 11-15
  5. >15

1. After you started providing community based palliative care did you notice an increase in the number of consultations/requests for help from the community?
   1. YES
   2. NO
2. Using the knowledge and experience you have gained; did you educate the community on community based palliative care?
   1. YES
   2. NO

If YES, please explain how you were able to do it?

____________________________________________________________________________________________________________________________________

1. What barriers do you experience while performing community based palliative care activities? (Pick as many that apply)
   1. I don’t feel my training is sufficient
   2. I don’t have enough time
   3. No access to expert opinion/support for consultation/teleconsultation
   4. It is expensive to visit households routinely
   5. Patients decline palliative care services
   6. Other (please specify)

_____________________________________________________________________

1. With the knowledge you gained on COVID 19, did you educate the community on the same?
   1. YES
   2. NO

If YES, did you notice any change in the behavior in the community?

- 1. YES
  2. NO

If YES, please explain how you were able to do it?

____________________________________________________________________________________________________________________________________

**Subjective assessment of level of knowledge pre- and post-training *(administered 12 months after the training)***

**Instructions: Kindly answer all the questions and select the level that you feel fits best** (1 being no knowledge and 5 being very knowledgeable)

1. My level of knowledge regarding COVID 19
   - Pre training 1 2 3 4 5
   - Post training 1 2 3 4 5
2. My knowledge regarding patient’s assessment of pain
   - Pre training 1 2 3 4 5
   - Post training 1 2 3 4 5
3. My knowledge regarding assessment of spiritual issues in patients
   - Pre training 1 2 3 4 5
   - Post training 1 2 3 4 5
4. My knowledge regarding on how to provide basic nursing care
5. Prevention and care of bedsores
   - Pre training 1 2 3 4 5
   - Post training 1 2 3 4 5
6. Feeding a disabled or bedbound patient
   - Pre training 1 2 3 4 5
   - Post training 1 2 3 4 5
7. Turning a disabled or bedbound patient on bed
   - Pre training 1 2 3 4 5
   - Post training 1 2 3 4 5
8. Cleaning and dressing of wounds
   - Pre training 1 2 3 4 5
   - Post training 1 2 3 4 5
9. Stoma care
   - Pre training 1 2 3 4 5
   - Post training 1 2 3 4 5
10. My knowledge on how to provide grief and bereavement care
    - Pre training 1 2 3 4 5
    - Post training 1 2 3 4 5
11. My knowledge on assessment of other physical symptoms like constipation, vomiting, fatigue, diarrhea, breathlessness
    - Pre training 1 2 3 4 5
    - Post training 1 2 3 4 5
12. My knowledge in application of non-medical interventions as part of care for other physical symptoms (constipation, vomiting, fatigue, diarrhea, breathlessness)
    - Pre training 1 2 3 4 5
    - Post training 1 2 3 4 5

**Semi-structured questions for Focus Group Discussion (FGD) *(administered 12 months after the training)***

**Instructions**

- Each training cohort of <12 participants is considered as one group for the FGD

1. Please describe to me how it has been for you to manage patient medication adherence?
2. Please describe to me what changes have occurred in your patient care practice since you have completed this training?
3. What part of the training was new to you?
4. How did the training change your practice?
5. How did you cope while seeing the very sick patients?
6. How do you think the community benefits from your training?
